# Italian version of the Rasch-Built Overall Amyotrophic Lateral Sclerosis Disability Scale (ROADS): validation and longitudinal performance

**DOI:** 10.1007/s00415-022-11483-3

**Published:** 2022-11-16

**Authors:** Andrea Fortuna, Daniele Sabbatini, Annachiara Frigo, Luca Bello, Francesca Calvi, Lorenzo Blasi, Giulia Gianferrari, Ilaria Martinelli, Giacomo Minicuci, Elena Pegoraro, Jessica Mandrioli, Gianni Sorarù

**Affiliations:** 1 Department of Neurosciences, Neuromuscular Center, University of Padova, 35128 Padua, Italy; 2 Department of Neurosciences, University of Padova, Padua, Italy; 3 Unit of Biostatistics, Epidemiology and Public Health, Department of Cardiac, Thoracic and Vascular Sciences, University of Padova, Padua, Italy; 4 Department of Neuroscience, Neurology Unit, S. Agostino Estense Hospital, AziendaOspedalieroUniversitaria di Modena, Modena, Italy; 5 Neuromuscular Center, S. Bortolo Hospital, Vicenza, Italy

**Keywords:** ALS, ROADS, ALSFRS-R, Italian, Scale, Validation

## Abstract

**Objective:**

To validate an Italian version of the Rasch-Built Overall ALS Disability Scale (ROADS) in a broad population of patients and assess its longitudinal performance over time.

**Methods:**

270 ALS patients referring to the Motor Neuron Disease Clinic of the University of Padova and Modena (Italy) accepted to compile the Italian version of the ROADS and results were correlated with the ALSFRSr and ALSAQ-40 scores, FVC values, and creatinine or albumin blood levels. To verify test–retest reliability, patients were asked to fill in a second copy of the scale within 5–7 days. Thirty-nine patients compiled a further copy of questionnaire during the follow up visit (after 133 days on average) which allowed us a longitudinal assessment of the scale.

**Results:**

We found a good external construct validity between ROADS and either ALSFRS-R (correlation coefficient = 0.85) or ALSAQ-40 (correlation coefficient = − 0.84). Test–retest reliability was excellent with a concordance-correlation coefficient of 0.93. Yet, we observed a significant correlation between changes over time of the ROADS normalised sum score (− 2.18 point loss per month) and those of both the ALSFRS-R (positive correlation; Rho = 0.64, *p* ≤ 0.0001) or the ALSAQ-40 (negative correlation; Rho = − 0.60, *p* = 0.014).

**Conclusions:**

The Italian version of ROADS proved to be a reliable marker to monitor overall disability in ALS patients. Further studies are necessary to assess its longitudinal performance.

**Supplementary Information:**

The online version contains supplementary material available at 10.1007/s00415-022-11483-3.

## Introduction

The Amyotrophic Lateral Sclerosis Functional Rating Scale (ALSFRS-R) is a widespread test used to evaluate progression of disability in ALS patients. It is commonly adopted in clinical trials as a primary outcome measure [1, 2], since it offers a reasonable correlation with other less accessible endpoints (survival, electrophysiologic measures, forced vital capacity, etc.) [3]. Nevertheless, the ALSFRS-R has also a series of weaknesses which hind its clinical and research utility, for instance the lack of responsiveness in a brief observation period or in advanced stages of the disease, and the presence of items that show improvement due to change in symptoms management [4]. Other important key points are the lack of linearity and unidimensionality [5–7].

Rasch-built scales are linearly weighted, meaning that 1-point change is a measurable unit across the scale, and unidimensional as all questions are measuring the same domain. The American Medical Association created and validated a Rasch-Built Overall ALS Disability Scale (ROADS), for use as a clinical outcome measure in patients with ALS [8]. A recent study assessed an Italian version of ROADS, pointing to the reliability of its administration to patients versus their respective caregivers and the correlation to the corresponding ALSFRS-R [9]. The goal of this study is to further validate the Italian version of the scale according to a test–retest methodology in a wider population of patients and also to examine its longitudinal performance over time.

## Methods

From July 2020 to March 2021, we consecutively proposed the compilation of the Italian Version of the ROADS to all patients with a diagnosis of ALS [10] referring to the Motor Neuron Disease Clinic of the University of Padova and the University of Modena. Comorbidity for dementia was considered an exclusion criteria. As for the original ROADS, response options to each item of the Italian version of the scale included 0 (unable to perform), 1 (able to perform but with difficulty or help), 2 (no abnormality). Patients were instructed to answer based on how they performed tasks before symptom onset, allowing caregivers to mark responses in case of patient’s inability to writing. A blank copy of the ROADS questionnaire was provided to be similarly completed at home within 5–7 days and returned to the study team by webmail after completion (test–retest analysis). Test–retest data were stratified for some possible interfering factors including: site of recruitment (University of Padova vs University of Modena), disease duration at questionnaire compilation (4 classes were considered: < 7 months, 7–13 months, 14–30 months, > 30 months), and presence of respiratory dysfunction (3 classes based on the ALSFRS-R item 10 score: 0–1, 2–3 points or 4 points). Along with the ROADS, the following outcome measures were collected as part of the routine follow-up protocol: the ALSFRS-R and the Amyotrophic Lateral Sclerosis Assessment Questionnaire – 40 (ALSAQ-40) scores [11], the Forced vital capacity (FVC) value (% of the predicted) [12], and serum creatinine and albumin levels [13]. To assess the longitudinal performance of the ROADS, patients evaluated twice during the study period were asked to fill in another copy of the questionnaire during the second visit. The time between the two visits was recorded.

A signed informed consent was obtained from all participants. The study was approved by the local Ethical Committees.

### Translations and cultural adaptation of the ROADS

The ROADS questionnaire was translated to Italian by two native Italian speakers fluent in English who have not seen the scale before. The Italian version was back-translated to English by two native English speakers fluent in Italian, who also have not seen the scale before. Adaptations were necessary to some of the 28 items of the scale, during the translation process, in consideration of cultural and language differences. Item 2, “ride in a car”, was easily misunderstandable, so we translated this item specifying “ride in a car as a passenger”. Since drinking milkshake or smoothies is not a widespread habit among Italians, item 4 was modified indicating a drink having the same consistency (changed to “drink a fruit juice”). For same reasons, item 8 was modified as “eat a cracker”. Italian translation of item 11, “eat a large meal”, raised the doubt between a sizeable meal and a full meal, so we changed it to “eat a full meal”.

### Statistical analyses

External construct validity was evaluated by assessing correlations of the ROADS normalised sum score (i.e. the raw sum score of the scale transformed into a linearly weighted score reflecting any 1-point change as a measurable and consistent unit of disability across the entire scale) [8] to ALSFRS-R, ALSAQ-40, serum creatinine, albumin and forced vital capacity (FVC). Correlation analysis was performed using Spearman’s correlation coefficients for ordinal data and Pearson correlation coefficient for continuous variables. Internal consistence was evaluated using Cronbach’s alpha test for ALSFRS-R, ROADS and ALSAQ-40 scores, setting the coefficient at 0 for null hypothesis. Lin's concordance test was used to assess the ROADS test–retest reliability considering the concordance correlation coefficient, CCC, and the confidence interval (CI) was calculated by the bootstrap method which is based on 2000 resamplings. For the two questionnaires, the first filled out at baseline and the second one completed 2–7 days apart, we considered either the overall normalised sum score and each single item score. We used the Bland–Altman graph for data plotting and the Wilcoxon paired test to compare the two ROADS questionnaire results. Finally, we applied the Spearman's rank correlation coefficient to compare the difference in ROADS normalised sum score averages between the first and second visit with ALSFRS-R and ALSAQ-40 score variations, considering the difference between the two visit values divided by the time elapsed in months. Significance was set at *p* < 0.05.

## Results

**Fig. 1:**
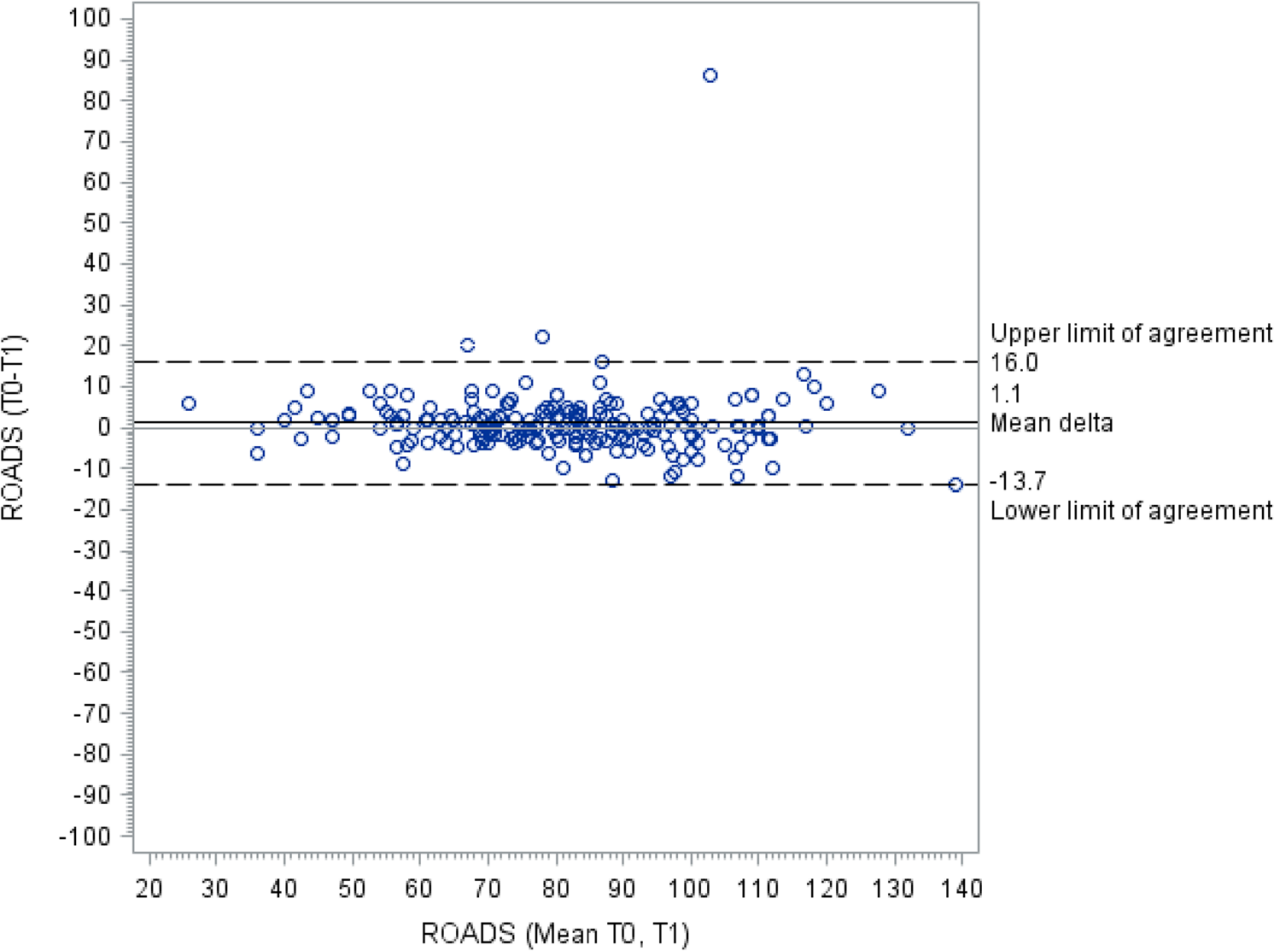
Bland-Altman graph showing a great test stability with short agreement limits. *ROADS* Rasch-Built Overall Amyotrophic Lateral Sclerosis Disability Scale. ROADS (T0-T1): difference between ROADS normalised sum score after 5-7 days and at baseline. ROADS (mean T0, T1): mean between ROADS normalised sum score after 5-7 days and at baseline

**Fig. 2:**
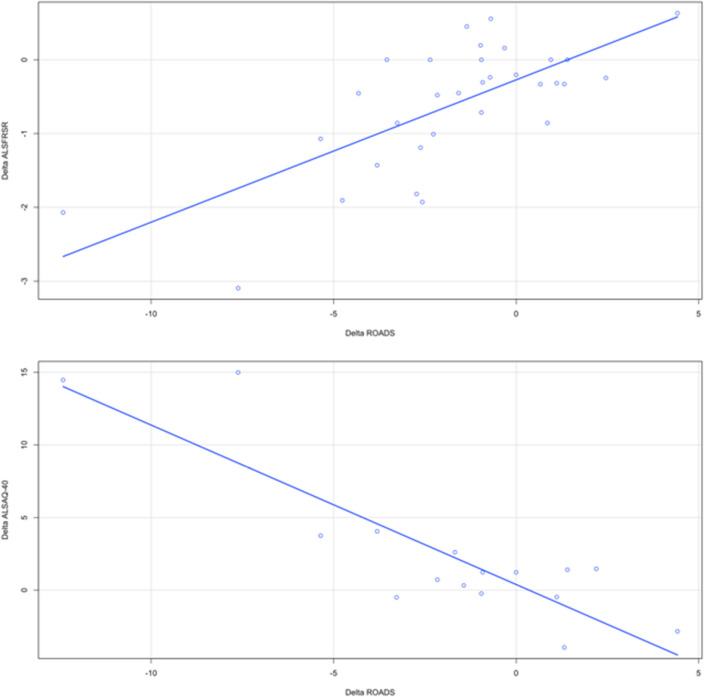
Scatter plot representing correlations between the mean differences of ROADS normalised sum scores between the first and the second visit and, respectively, ALSFRS-R and ALSAQ-40 sum scores’ variations. We can observe a great positive correlation between ROADS and ALSFRS-R sum scores and a good inverse correlation between ROADS and ALSAQ-40. *ROADS *Rasch-Built Overall Amyotrophic Lateral Sclerosis Disability Scale. ALSFRS-R: Amyotrophic Lateral Sclerosis Functional Rating Scale. ALSAQ-40: Amyotrophic Lateral Sclerosis Assessment Questionnaire – 40

A total of 270 ALS patients accepted to fill in the ROADS questionnaire during one formal visit in hospital, although 38 missed to return the other copy of the scale. For each patient, all the other functional measures were also recorded. Characteristics of total cohort of patients are shown in Table 1S. We found a good external construct validity between ROADS and either ALSFRS-R or ALSAQ-40 (Fig. 1S), with a correlation of 0.85 (CI [0.81; 0.88], *p* < 0.0001) and − 0.84 (CI [− 0.88: − 0.80], *p* < 0.0001), respectively. Neither serum creatinine (correlation coefficient 0.44, CI [0.29; 0.57], *p* < 0.0001) nor albumin (correlation coefficient 0.26, CI [0.04; 0.45], *p* = 0.0094) showed a good correlation with the ROADS score, whereas FVC values mildly correlated (correlation coefficient 0.57, CI [0.44; 0.68], *p* < 0.0001). Cronbach’s alpha showed a good internal consistency for the ROADS (*α* = 0. 9548) as well for the ALSFRS-R (*α* = 0.8891) and the ALSAQ-40 (*α* = 0.9602) scales. Test–retest reliability, evaluated from those 232 patients who completed both ROADS copies, was excellent (CCC = 0.93; CI [0.77; 0.97]). The mean test value was 82.40 (standard deviation = 20.29) and the mean retest value 81.25 (standard deviation = 20.68), with a bias of 1.15 (standard deviation = 7.6), resulting in a *p* value of 0.026 with Wilcoxon paired-test. Analogous results were obtained also considering each single item. Likewise, Bland–Altman graph showed low dispersion with short agreement limits (Fig. 1) and concordance–correlation analysis for every single item showed great stability. None of the considered confounding factors showed to interfere with test–retest analysis (Figs. 2, 3 and 4S).

Thirty-nine patients, among those who were evaluated twice during the study period, filled in a further copy of the ROADS questionnaire during the follow up visit. The mean interval between their two visits was of 144 days (median 133 days). By comparing data collected at the first and the second visit (follow-up visit), we could observe a significant correlation between changes of the ROADS normalised sum score and those of both ALSFRS-R (positive correlation; Rho = 0.64; *p* < 0.0001) or ALSAQ-40 (negative correlation; Rho = − 0.60; *p* = 0.014) (Fig. 2). The ROADS normalised sum score declined by 2.18 point per month on average. Longitudinal FVC or creatinine and albumin data were insufficient for a proper statistical analysis.

## Discussion

We further investigated the performance of the Italian ROADS version in a broader sample of ALS patients and considered the level of response agreement administering the scale to each patient in two different times. In full accordance with the previous Italian study [9], we confirmed both an excellent internal and external (by comparison with the most widely used ALSFRS-R) consistency of our scale version. Although the intra-patient test–retest analysis returned an excellent concordance coefficients for each item responses including those related to the most challenging actions, an up to 16-point variability of the scale score was observed in some patient. This emphasizes the need to be accurate in explaining to patients how to exactly answer to the questionnaire. Our findings resulted unchanged after splitting cases into the two cohorts of patients, i.e. Padova and Modena, involved in the study and neither disease duration or respiratory functioning showed to affect consistency of answers.

Similarly, the setting where the questionnaire was compiled, i.e. hospital or home, appeared to have no bearing on the type of response. This is quite interesting as it implies that ROADS could be reliably used also for remote patient monitoring and thus it may represent a strategic tool for the ordinary clinical practice or in the context of clinical trials.

To keep the same conditions as the original version of the questionnaire, we had to modify the translation of some questions. Nevertheless, translation changes did not affect items functioning. In any case, we agree with the Chinese team [14] that a vignette representing the function explored by each question could help patients in completing/fulfilling the scale.

The novelty of our study is the longitudinal assessment of the ROADS which pointed to a stable correlation of the scale with progression of disability measured by the ALSFRS-R and quality of life according to the ALSAQ-40. It would have been interesting to compare the sensitivity as progression measure of the ROADS with ALSFRS-R being both functional scales; however, the small number of patients evaluated twice along with the short average time elapsed between the first and the second visit prevented us from drawing definite conclusions.

Finally, the Italian version of ROADS proved to be a reliable marker to monitor overall disability in ALS patients. Notwithstanding, further longitudinal studies are necessary to establish the ability of the scale to intercept disease changes over time even compared to measures already in use.

## Supplementary Information

Below is the link to the electronic supplementary material.

Supplementary file1 (DOCX 832 KB)
