# Correction to: Italian version of the Rasch-Built Overall Amyotrophic Lateral Sclerosis Disability Scale (ROADS): validation and longitudinal performance

**DOI:** 10.1007/s00415-023-11620-6

**Published:** 2023-02-22

**Authors:** Andrea Fortuna, Daniele Sabbatini, Annachiara Frigo, Luca Bello, Francesca Calvi, Lorenzo Blasi, Giulia Gianferrari, Ilaria Martinelli, Giacomo Minicuci, Elena Pegoraro, Jessica Mandrioli, Gianni Sorarù

**Affiliations:** 1 Department of Neurosciences, Neuromuscular Center, University of Padova, 35128 Padua, Italy; 2 Department of Neurosciences, University of Padova, Padua, Italy; 3 Unit of Biostatistics, Epidemiology and Public Health, Department of Cardiac, Thoracic and Vascular Sciences, University of Padova, Padua, Italy; 4 Department of Neuroscience, Neurology Unit, S. Agostino Estense Hospital, Azienda Ospedaliero Universitaria di Modena, Modena, Italy; 5 Neuromuscular Center, S. Bortolo Hospital, Vicenza, Italy; 6 Department of Biomedical, Metabolic and Neural Sciences, University of Modena and Reggio Emilia, Modena, Italy

**Correction to: Journal of Neurology** 10.1007/s00415-022-11483-3

The original version of this article unfortunately contained a mistake. The affiliation details for Author ‘’Jessica Mandrioli’’ were incorrectly given as ‘’Neuromuscular Center, S. Bortolo Hospital, Vicenza, Italy’’ but should have been:

Department of Biomedical, Metabolic and Neural Sciences, University of Modena and Reggio Emilia, Modena, Italy

Department of Neuroscience, Neurology Unit, S. Agostino Estense Hospital, Azienda Ospedaliero Universitaria di Modena, Modena, Italy

