# Supplementary material for: Italian version of the Rasch-Built Overall Amyotrophic Lateral Sclerosis Disability Scale (ROADS): validation and longitudinal performance
Source: J Neurol. 2022 Nov 16;270(3):1452–6. doi: 10.1007/s00415-022-11483-3 (PMC9971085; doi:10.1007/s00415-022-11483-3)
Supplement: Supplementary file 1 — Supplementary file1 (DOCX 832 KB) [file 415_2022_11483_MOESM1_ESM.docx]

**Italian version of the Rasch-Built Overall Amyotrophic Lateral Sclerosis Disability Scale (ROADS): validation and longitudinal performance**

**SUPPLEMENTAL MATERIAL**

**Table 1.** Demographic and clinical characteristics of questionnaire study participants.

FTD: Frontotemporal dementia

| **Characteristic** | **Mean** |
| --- | --- |
| **Age at onset** | 57.0 years |
| **Age at enrollment** | 23.7 months |
| **Diagnostic delay** | 23.7 months |
| **Sex Category** | **n (%)** |
| Male | 157 (58,15%) |
| Female | 113 (41,85%) |
| **Site of Onset** | **n (%)** |
| Missing | 7 (2,59%) |
| Bulbar | 42 (15,56%) |
| Lower limb | 130 (48,15%) |
| Upper limb | 74 (27,41%) |
| Bulbar + Lower limb | 2 (0,74%) |
| Bulbar + Upper Limb | 2 (0,74%) |
| Upper limb + Lower limb | 8 (2,96%) |
| Respiratory | 5 (1,85%) |
| **Behavioural changes (in FTD spectrum)** | **n (%)** |
| Missing | 15 (5,56%) |
| No | 226 (83,70%) |
| Yes | 29 (10,74%) |
| **Cognitive changes (in FTD spectrum)** | **n (%)** |
| Missing | 16 (5,93%) |
| No | 231 (85,56%) |
| Yes | 23 (8,52%) |

**Figure 1S.** Scatter plot representing correlations at baseline between ROADS raw sum scores, ROADS normalised sum scores, ALSFRS-R and ALSAQ-40. Both ALSFRS-R and ALSAQ-40 demonstrated a good correlation at baseline with ROADS.


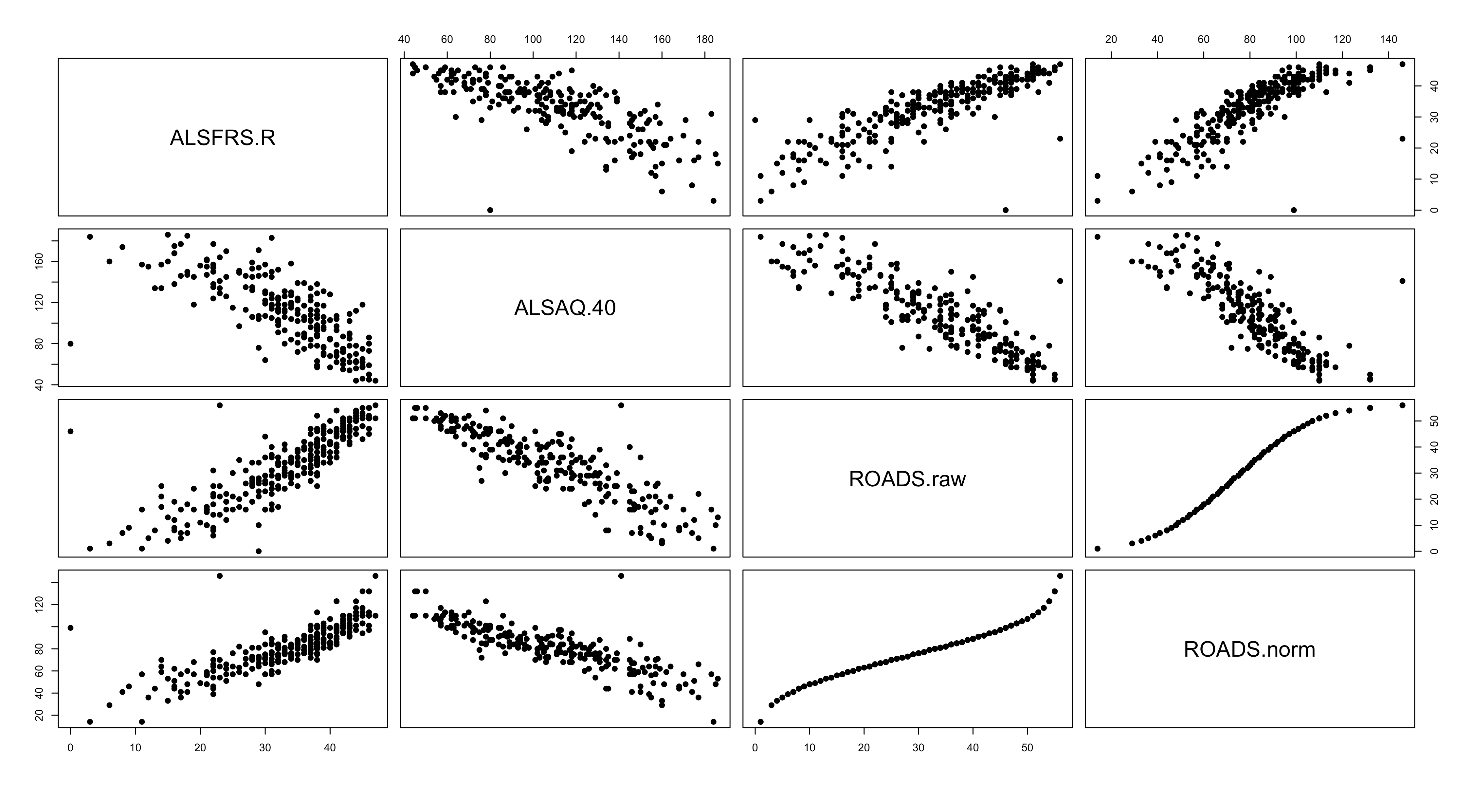


ROADS*:* Rasch-Built Overall Amyotrophic Lateral Sclerosis Disability Scale*.* ALSFRS-R: Amyotrophic Lateral Sclerosis Functional Rating Scale. ALSAQ-40: Amyotrophic Lateral Sclerosis Assessment Questionnaire – 40.

**Figure 2S.** Bland-Altman graph showing a great test stability with short agreement limits both for Padova’s (A) and for Modena’s group (B).

**
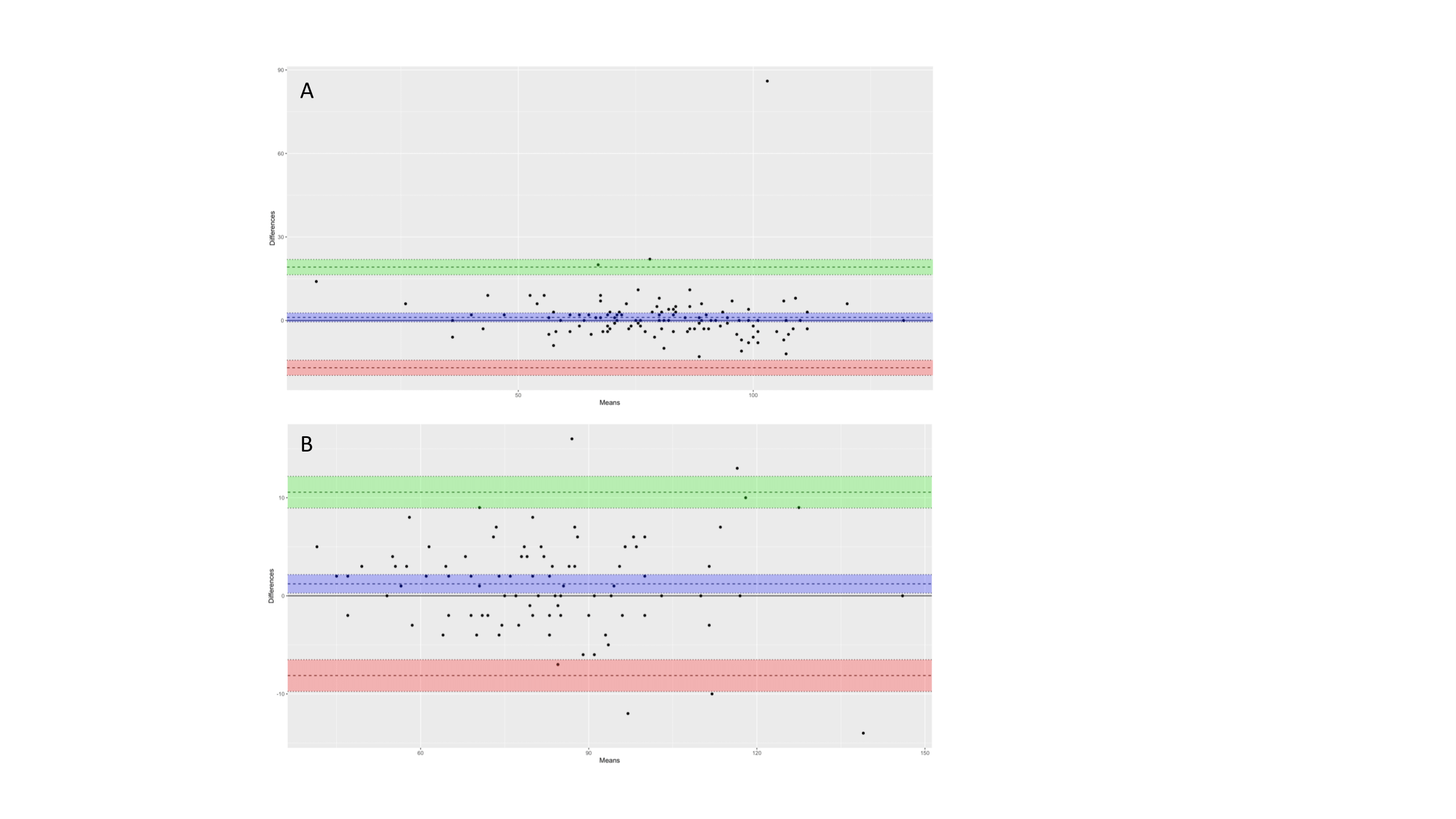
**

Differences: differences between ROADS normalised sum score after 5-7 days and at baseline. Means: mean between ROADS normalised sum score after 5-7 days and at baseline.

**Figure 3S.** Bland-Altman graph showing a great test stability with short agreement limits dividing the sample into four classes considering diagnostic delay: <7 months (A), between 7 and 13 months (B), between 13 and 30 months (C), >30 months (D).


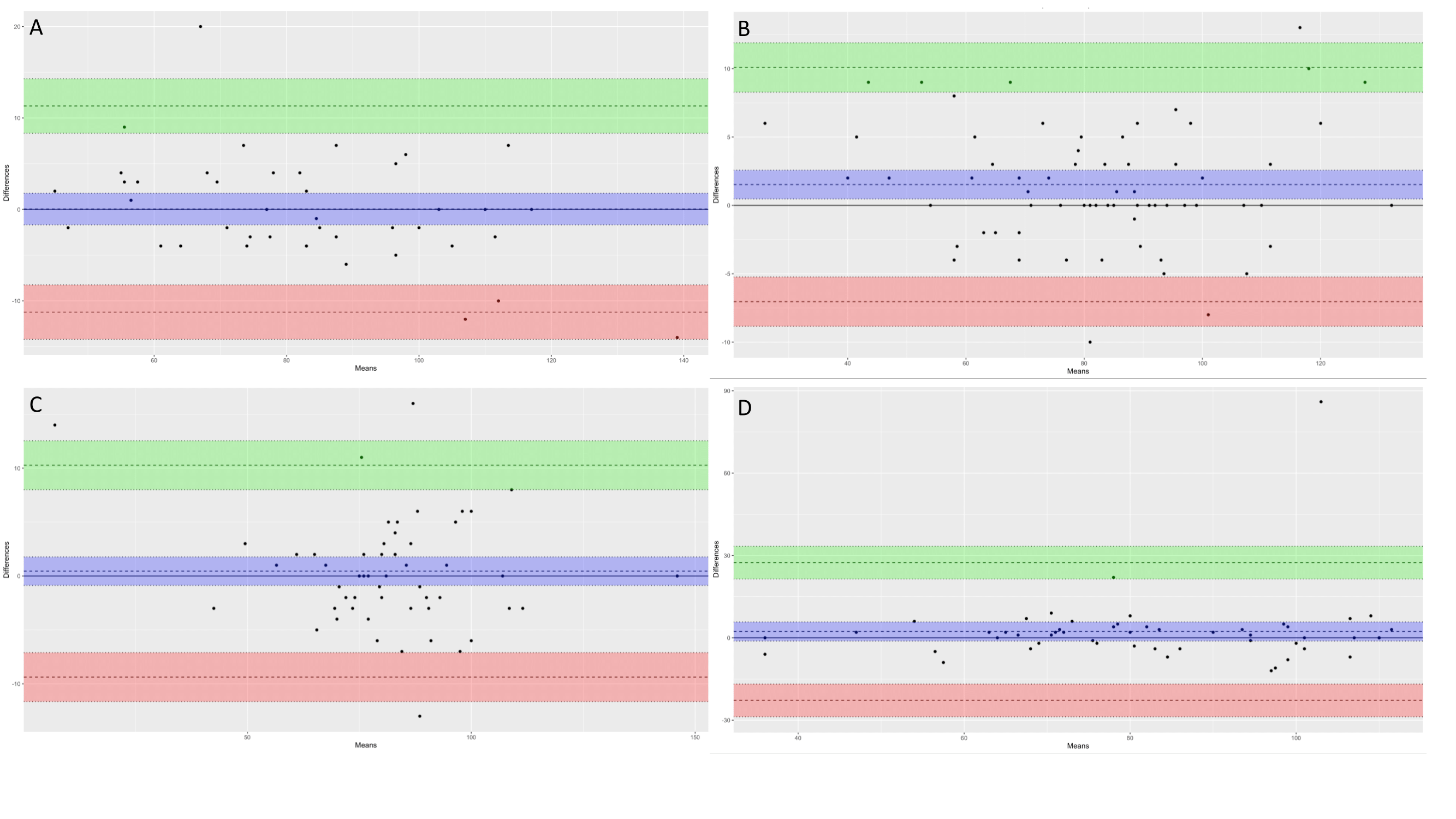


Differences: differences between ROADS normalised sum score after 5-7 days and at baseline. Means: mean between ROADS normalised sum score after 5-7 days and at baseline.

**Figure 4S.** Bland-Altman graph showing a great test stability with short agreement limits dividing the sample into three classes considering presence of dyspnoea (item 10 of ALSFRS-R): 0-1 points (A), 2-3 points (B), 4 points (C).


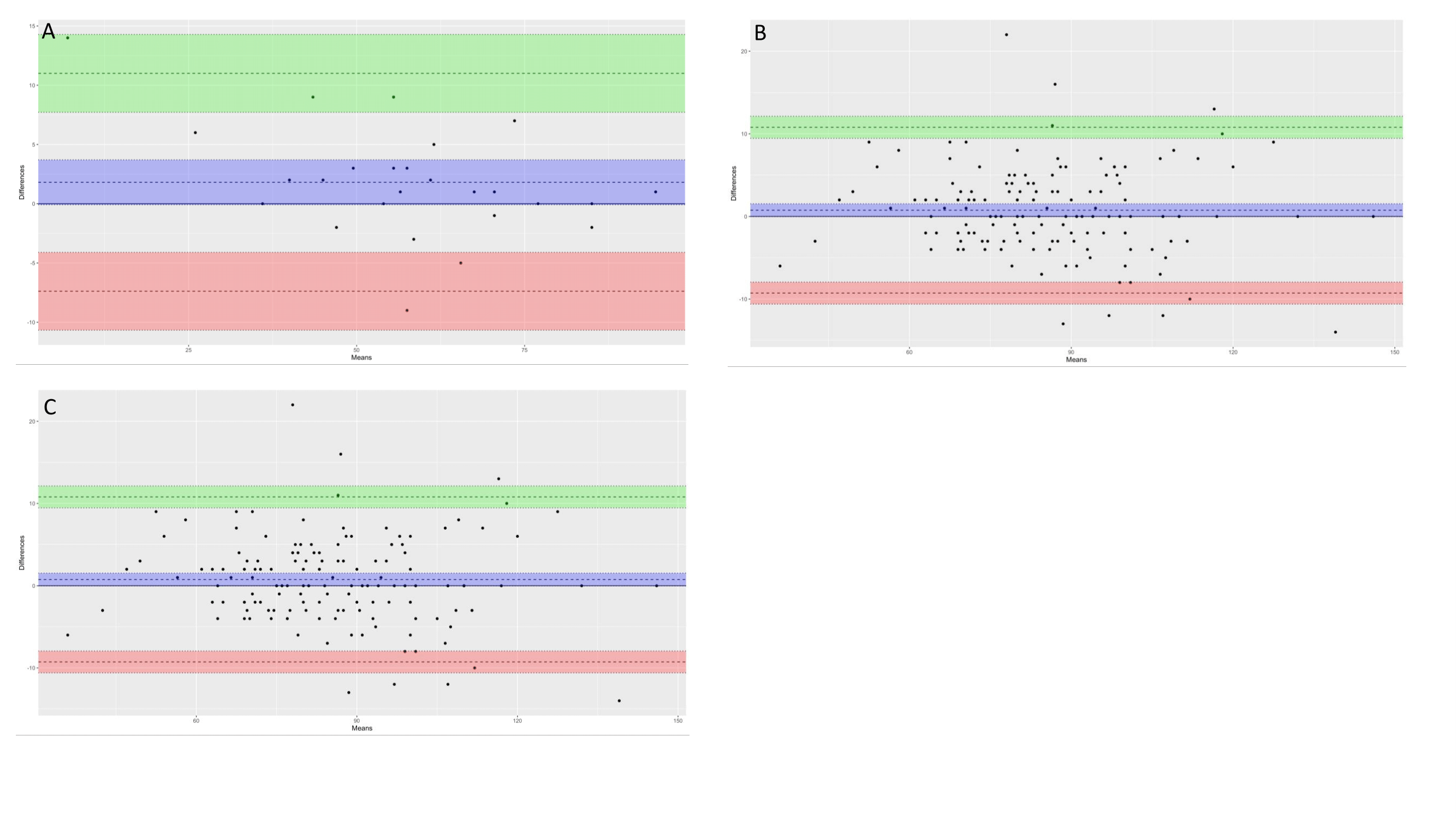


Differences: differences between ROADS normalised sum score after 5-7 days and at baseline. Means: mean between ROADS normalised sum score after 5-7 days and at baseline.
